# The Prison and Transition Health (PATH) Cohort Study: Study Protocol and Baseline Characteristics of a Cohort of Men with a History of Injecting Drug Use Leaving Prison in Australia

**DOI:** 10.1007/s11524-019-00353-5

**Published:** 2019-04-15

**Authors:** Amy Kirwan, Michael Curtis, Paul Dietze, Campbell Aitken, Emma Woods, Shelley Walker, Stuart Kinner, James Ogloff, Tony Butler, Mark Stoové

**Affiliations:** 1 Behaviours and Health Risks Program, Public Health Discipline, Burnet Institute, 85 Commercial Rd, Melbourne, VIC 3004 Australia; 2 School of Public Health and Preventive Medicine, Monash University, Melbourne, Australia; 3 National Drug Research Institute, Curtin University, Perth, Western Australia; 4 Centre for Adolescent Health, Murdoch Children’s Research Institute, Melbourne, Australia; 5 Centre for Mental Health, Melbourne School of Population and Global Health, University of Melbourne, Melbourne, Australia; 6 Griffith Criminology Institute, Griffith University, Brisbane, Australia; 7 Mater Research Institute-UQ, University of Queensland, Mount Gravatt, Australia; 8 Centre for Forensic Behavioural Science, Swinburne University of Technology and Forensicare, Melbourne, Australia; 9 Kirby Institute, University of New South Wales, Sydney, Australia

**Keywords:** Prison, Injecting drug use, Cohort study

## Abstract

People who inject drugs (PWID) are disproportionately represented among individuals who experience imprisonment and often have more complex physical and mental health needs than people in prison without injecting histories. The trajectories of PWID after prison release are poorly understood, hampering the development of effective strategies to address their distinct health needs. The Prison and Transition Health (PATH) Cohort Study is characterising the post-release trajectories of incarcerated male PWID in Victoria, Australia. We outline study methodology and baseline characteristics of participants prior to their release. Four hundred participants were recruited from three prisons and completed researcher-administered baseline interviews covering socio-demographics, social supports, physical health, mental health, alcohol and other drug use, and pre-release and transitional service utilisation. The median age among participants was 36 years (IQR 30–42), and they reported a median of five (IQR 3–9) previous adult incarcerations. Almost half (49%) were reliant on government payments prior to incarceration. One quarter (25%) of participants reported removal from their parents’ care as children and 64% reported being a parent or primary caregiver to children. Most participants (81%) reported a previous mental health diagnosis and 44% reported three or more diagnoses. The most common drugs injected prior to incarceration were crystal methamphetamine (80%) and heroin (62%), and most (85%) reported being under the influence of drugs at the time of committing offences for which they were currently incarcerated. Injecting drug use during their current sentence was reported by 40% of participants, and 48% reported engaging with some form of drug treatment during their current sentence. Study participants are characterised by significant mental health and substance use morbidities, social disadvantage and criminogenic histories that present challenges for the provision of post-release support services. Data from the PATH Cohort Study will help inform strategies to improve the health and social outcomes of this population.

## Background

People who inject drugs (PWID) are disproportionately represented among those who experience imprisonment [1]. Among people incarcerated in Australia, approximately half report ever injecting drugs [2] and approximately one quarter report injecting in the month prior to prison reception [3, 4]. Prisoners have considerably more complex physical and mental health needs [2, 3, 5, 6] and experience greater economic, psycho-social and educational disadvantage than the general population [7]. Those with a history of injecting drug use (IDU) have increased risk of a range of additional adverse health outcomes, including blood-borne virus (BBV) infections [2, 4, 8] and overdose [9, 10]. In Australia, these overlapping risks disproportionately affect Aboriginal and/or Torres Strait Islanders, who are over-represented in prison populations [11, 12], more likely to use injectable illicit drugs [13], and also experience greater socio-economic disadvantage and earlier contact with the criminal justice system [14].

Challenges associated with transitioning from prison to the community, including financial and relationship stress, social isolation and stigma, can be heightened for people with histories of drug dependence and mental and physical health comorbidities [15–17] and contribute to post-release service access barriers [18]. For those with drug dependence histories, return to drug use [19] and recidivism [20] following release and reincarceration are also common. Approximately 40% of all people released from prison in Australia return within 2 years [21], with 2-year reincarceration rates among those with IDU histories estimated to be as high as 84% [22].

Extensive literature describes the health and social challenges of people released from custody; however, the trajectories of people released from prison who were engaging in regular IDU in the months immediately prior to incarceration remain poorly understood. Given their health and social vulnerabilities and high rates of recidivism, the scarcity of studies describing the post-release natural history of this group represents a significant gap. To date, the few cohort studies of people released from prison in Australia have provided limited insights given they recruited general (i.e. unselected) prisoner populations [23, 24], relied exclusively on record linkage [22, 25, 26], recruited small samples with short follow-up [15, 20], or recruited in the community in the weeks after release [27]. These studies also lacked the depth of information needed to detail the temporal relationships between incarceration, IDU, physical and mental health, health service utilisation and recidivism.

The Prison and Transition Health (PATH) Cohort Study aims to characterise the prison-to-community trajectories of incarcerated males in Victoria, Australia, who report regular IDU in the months immediately prior to incarceration. This paper outlines the PATH Cohort Study research protocol and describes the baseline (pre-release) characteristics of the sample.

## Methods

### Study Design

This prospective cohort study recruited incarcerated males nearing the end of their sentences who had a self-reported history of regular IDU in the months immediately prior to incarceration. Data collection consisted of in-depth quantitative interviews, blood specimen collection and blood-borne virus testing, and record linkage to health, housing and justice databases. Primary data collection for the study included extensive quantitative interviews conducted at baseline (pre-release) and 3, 12 and 24 months after release, which constitute the primary study data. Blood specimen collection occurred at baseline, 12 months and 24 months. Data linkage is planned at 2, 5 and 10 years post-release to describe long-term service interactions and health, social and criminal justice outcomes. This paper describes the sample on the basis of their responses to baseline interviews which were conducted in prison.

### Setting

Between September 2014 and June 2016, participants were recruited from three male prisons in the Australian state of Victoria. Operational limitations in the state’s women’s prisons at the time of data collection prevented planned recruitment of female participants. Recruitment prisons were selected to enhance sample heterogeneity by geography (metropolitan, regional) and security level (minimum, medium, maximum security). Recruitment targets at each prison aimed to reflect a proportional representation of the prison population size at each site. The male prisoner population in Victoria in 2016 was estimated at 6,644 [28] and the minimum, medium and maximum security recruitment prisons accommodated an estimated 242, 552 and 1067 prisoners, respectively [29].

### Eligibility Criteria

Individuals were eligible to participate if they self-reported injecting drugs at least once a month in the 6 months before incarceration, were being aged 18 years or over,^1^ were a sentenced prisoner (i.e. not on remand), consented to participate in four face-to-face interviews (in-prison baseline and three follow-up interviews in the community), provide blood specimens, be regularly contacted by researchers over the follow-up period and record linkage for 10 years following recruitment.

Baseline interviews were scheduled for within 12 weeks of participants’ expected release dates, which were determined using a combination of recorded end-of-sentence dates and prisoner self-report of anticipated early release on parole, validated by prison staff at the time of recruitment. When release was delayed 12 weeks beyond baseline interviews, short catch-up interviews were conducted to update time-variant pre-release data.

### Recruitment and Consent

Recruitment criteria of recent pre-incarceration IDU prevented random sampling using prison administrative records. To recruit participants, researchers engaged directly with prisoners at medical centres during dosing times for opioid substitution therapy (OST), at alcohol and other drugs (AOD) therapeutic group sessions, through prison workplaces and in prison units/cell blocks. Researchers engaged peer representatives and staff involved in program delivery to prisoners with IDU histories to promote the study and presented at ‘town hall meetings’ (regular meetings of prisoners and staff), educational programs and prison workplaces. Study posters were displayed in general access areas of prisons. Those interested in participating who were not engaged directly by researchers completed ‘expression of interest’ forms, which they could submit directly to researchers, clinical or program staff, or place in secure mailboxes in prisons. These forms helped protect confidentiality and provided consent for researchers to contact prisoners and discuss the study in accordance with prison operations and ethics committee protocols. Researchers screened potential participants for eligibility, and once eligibility was established, a baseline interview was scheduled.

During the informed consent process, researchers verbally reinforced key elements of the written participant information and consent forms. The concept and process of record linkage, including participant confidentiality, and types of information sought from data custodians, was discussed with participants. In accordance with government requirements, participants completed a separate form to consent to data linkage to their federal medical records. Pre and post-test discussions occurred with all participants when baseline blood samples were collected, with test results delivery scheduled following release.

### Baseline Data Collection

Researchers used electronic tablets to administer quantitative questionnaires, and data were downloaded into an electronic database (Mobile Data Studio Software) [30]. Questionnaires covered six domains: socio-demographics, social support, physical health, mental health, alcohol and other drug use and related behaviours, and pre-release and transitional services and arrangements. Items were adapted from those used in a cohort study of community-recruited PWID [31] and tested in a pilot study [20], alongside standardised and validated scales (Table 1). Mean duration of interviews was 45 min (SD = 12 min, range 26–73 min).

**Table 1:** Summary of variables collected at baseline interview

Domain	Content
Socio-demographics	Date of birth
	Country of birth
	Language spoken
	Aboriginal and Torres Strait Islander status
	Sexual identity
	Relationship status
	Accommodation status
	Children
	Level and type of education completed
	Income prior to prison
	Employment prior to prison
Social support	Recent contact with family and friends
	Availability of social supports
	Family history
Physical health	Current health status
	Physical illnesses/conditions
	Viral infections (status, testing and treatment)
	Medications
	Disabilities and injuries
Mental health	Current mental health status (GHQ-12) [46]
	Mental health service usage
	Diagnoses
	Suicide and self-harm
Alcohol and other drugs and related behaviours	Alcohol use prior to prison (AUDIT C) [47]
	Licit and illicit drug use prior to and in prison
	Injecting drug use (history, risks, behaviours)
	Use of OST
	Use of other AOD services and programs
	Overdose
	Level of Service Inventory-Revised: Screening Version (LSI-R:SV) [48]
Pre-release and transitional services and arrangement	Use of programs and services
	Plans for release
	Concerns about release
	Expectations after release

A dry blood spot finger-prick blood specimen was collected for hepatitis C antibody testing using commercially available assays. HIV testing was not performed due to the low prevalence of HIV among Victorian prison populations [4]. Hepatitis B testing was not performed due to budget limitations and the cost of dry blood spot assays.

Contact-tracing details were collected on paper-based forms and stored in an electronic database separate to survey data. To facilitate follow-up, researchers collected detailed participant identifying information (full name, date of birth, alias/street name, expected residential address, expected telephone numbers after release) and secondary contact details for friends, relatives, services or specific workers they anticipated contacting after release.

Participants were not reimbursed for baseline interviews in prison in accordance with the Corrections Victoria guidelines.

### Follow-up Data Collection

Researchers are conducting follow-up interviews at 3, 12 and 24 months after release from prison (3- and 12-month interviews complete at time of writing) and collecting venous blood samples for hepatitis B and hepatitis C antibody and virus testing (including RNA tests to determine chronic/active infections) at 12 and 24 months. Researchers provide blood test results to participants where possible and offer referral to hepatitis care and treatment providers.

Follow-up questionnaires are adapted from those administered at baseline, removing time-invariant items and adding or adapting items to capture prospectively occurring events and to reflect differences between the community and prison environments. Participants receive AUD40 as a cash payment for each follow-up interview completed in the community.

Researcher contact with participants during follow-up occurs via regular phone, email and social media contact, or via secondary contacts described above. Researchers also receive information on whether a participant who cannot be contacted has been reincarcerated during follow-up. Follow-up protocol permitted interviews to occur in any prison in Victoria in these circumstances. At the time of writing, all three waves of follow-up had greater than 50% participation and more than 80% of participants had completed at least one follow-up interview.

### Record Linkage

Record linkage will be conducted at 2, 5 and 10 years post-release across the following health and justice databases: Medicare and the Pharmaceutical Benefits Scheme (federally funded healthcare), state-wide mental health, alcohol and other drug treatment, ambulance, hospital emergency department, hospital admissions, housing services, police contact (arrest, charge, victim) and mortality. Participants consented to data linkage on prison program participation, including those related to addressing offending behaviour, drug use and use of prison health services, during the sentence in which they were recruited and future periods of incarceration over 10 years. Record linkage had not commenced at the time of writing.

### Ethics Approval

The Victorian Department of Justice Human Research Ethics Committee and the Alfred Hospital Ethics Committee approved the study. Specific ethical and administrative approvals for record linkage were received from the Australian Government Department of Health, the Australian Institute of Health & Welfare, Victoria Police, and the National Coronial Information System.

### Analysis

In this paper, we present descriptive statistics to characterise the socio-demographics, physical and mental health, incarceration history, pre-incarceration offending, drug dependence treatment history and substance use of participants. These were generated using Stata SE Version 14.1 [32].

## Results

Four hundred and nine participants were recruited over 21 months. Recruitment proved challenging within the prison setting due to various issues, including negotiating access to locations where researchers could engage potential participants directly, building trust between researchers and potential participants and transfer of prisoners out of recruitment prisons to other sites prior to scheduled interviews occurring.

Data from nine participants were excluded; six had lengthy delays in release dates (still in prison at time of initial data analysis), two revealed at post-release interviews they had not met pre-incarceration IDU eligibility criteria and one due to a technical error in data collection. The resultant sample of 400 participants included 108 (27%) recruited from low-security, 111 (28%) from medium-security and 181 (45%) from high-security prisons. Participants’ median sentence length was 206 days (IQR 109–381), and the median time between baseline interview and release from prison was 33 days (IQR 13–62 days).

### Demographics

The median age of participants was 36 years (IQR 30–42). Seventeen percent of participants identified as Aboriginal and/or Torres Strait Islander and 90% were born in Australia. Most participants (83%) had not completed high school, and approximately half (49%) reported government payments as their primary source of income prior to incarceration. Twenty-eight percent of participants reported living in unstable accommodation in the 6 months prior to incarceration. A quarter of participants (25%) reported being removed from parents’ care as children. Almost two thirds (64%) reported being a parent or primary caregiver to a child/ren, and of these, almost half (46%) reported involvement of child protection and one in five (20%) reported having ever had children removed from their care (Table 2).

**Table 2:** Socio-demographics and pre-incarceration offending characteristics at baseline

Variable	*N* = 400
	*n* (%)
Socio-demographics	
Median age (years)(IQR)	36 (30–42)
Aboriginal or Aboriginal and Torres Strait Islander	66 (17)
Australian born	358 (90)
English as primary language spoken at home	388 (97)
Highest education level completed	
Year 7–9^1^	168 (42)
Year 10–11	162 (41)
Year 12/completed secondary school^2^	36 (9)
Certificate/Diploma	34 (9)
Main income source before prison	
Government payment^3^	197 (49)
Illegal activities	154 (39)
Paid work (inc. cash in hand)	36 (9)
Other sources^4^	9 (4)
Accommodation type before prison	
Private rental (single or shared)	113 (28)
Family member’s home	80 (20)
Public housing	73 (18)
Temporary accommodation^5^	57 (14)
No fixed address/car/squat	36 (9)
Owner occupied	23 (6)
Self-reported accommodation as unstable before prison	111 (28)
Ever removed from parent’s care	100 (25)
Parent or caregiver	257 (64)
Child protection involvement with children^6^	118 (46)
Children removed from your care^6^	51 (20)
Incarceration and pre-incarceration offending	
Median self-reported adult incarceration episodes (IQR)	5 (3–9)
Incarcerated as a Juvenile	179 (45)
Under the influence of alcohol or other drugs at the time of committing this offence?	
Yes, drugs only	237 (59)
Yes, drugs and alcohol	104 (26)
Yes, alcohol only	17 (4)
Current sentence related to purchase drugs or alcohol	156 (40)

^1^Includes 13 who did not complete any high school education

^2^Includes 10 who completed tertiary education

^3^Government payments include unemployment benefits, study benefits and pensions

^4^Other sources include money from family and friends, withdrawn savings and begging

^5^Temporary accommodation includes staying with friends, boarding houses and crisis accommodation

^6^Among those reporting to be parents or caregivers

### Incarceration and Pre-Incarceration Offending History

The median number of reported prior adult incarcerations was five (IQR 3–9), and 179 (45%) participants reported being incarcerated at least once as a juvenile. Most participants (85%) reported being under the influence of drugs (drugs alone or in combination with alcohol) at the time of committing the offence(s) for which they were currently imprisoned. Almost half (40%) reported their offending occurred because they needed money to purchase alcohol or other drugs (Table 2).

### Physical and Mental Health

Infectious diseases were the most commonly reported general health condition (75%); 74% self-reported a previous hepatitis C diagnosis, and confirmatory seroprevalence of hepatitis C antibodies was 82%. Seven percent of participants self-reported a previous diagnosis of hepatitis B, and less than 1% self-reported a diagnosis of HIV. Other commonly reported health conditions included dental (70%), musculoskeletal (45%) and respiratory (31%) health conditions. One in five participants (20%) reported having been diagnosed with an acquired brain injury. Most participants (83%) reported a previous mental health diagnosis. Almost half (44%) reported three or more mental health diagnoses. Depression (66%), anxiety (50%) and drug-induced psychosis (34%) were the most common mental health diagnoses (Table 3).

**Table 3:** Health and mental health characteristics at baseline

Variable	*N* = 400
	*n* (%)
Self-reported physical health conditions	
Chronic infectious disease	298 (75)
Ever tested positive for hepatitis C	293 (74)
DBS hepatitis C antibody prevalence	328 (82)
Dental condition	281 (70)
Musculoskeletal condition	180 (45)
Respiratory disease	122 (31)
Hearing or vision condition	100 (25)
Neurological disease	48 (12)
Circulatory disease	46 (12)
Metabolic disease	13 (3)
Self-reported mental health conditions	
Any mental health condition	332 (83)
Three or more mental health conditions	175 (44)
Depression	262 (66)
Anxiety disorder	201 (50)
Drug-induced psychosis	137 (34)
Post-traumatic stress disorder	85 (21)
Bipolar disorder	66 (17)
Personality disorder^1^	63 (16)
Schizophrenia	56 (14)
Panic disorder	54 (14)
Other mental health disorder^2^	47 (12)

^1^Includes anti-social, borderline and other personality disorders

^2^Includes Schizoaffective disorder, attention deficit hyperactivity disorder, adjustment disorder, autism spectrum disorder, disorder, eating disorder, obsessive compulsive disorder and paedophilia

### Substance Use

The median age of reported first episode of IDU was 17 years (IQR 15–20). The most commonly used substances by any route of administration and injected in the month prior to incarceration were crystal methamphetamine (79% and 74%, respectively), heroin (57% and 55%), benzodiazepines (31% and 2%) and prescription opioids (20% and 18%) (Table 4).

**Table 4:** Illicit drug use characteristics at baseline

Illicit substance*	Ever used	Used month before incarceration	Ever injected	Injected month before incarceration
	*n* (%)	*n* (%)	*n* (%)	*n* (%)
Cannabis	374 (94)	258 (65)	NA	NA
Crystal methamphetamine	372 (93)	315 (79)	361 (90)	296 (74)
Heroin	355 (89)	226 (57)	344 (86)	220 (55)
Speed	355 (89)	75 (19)	329 (82)	72 (18)
Ecstasy	286 (72)	33 (8)	132 (33)	11 (3)
Cocaine	277 (69)	52 (13)	168 (42)	28 (7)
Prescription opiates	273 (68)	81 (20)	242 (61)	72 (18)
Benzodiazepines	259 (65)	122 (31)	55 (14)	9 (2)
Buprenorphine (Subutex and Suboxone)	225 (56)	49 (12)	176 (44)	31 (8)
Methadone	126 (32)	19 (5)	26 (7)	2 (1)
Pharmaceutical stimulants	122 (31)	18 (5)	61 (15)	13 (3)

*Illicit substances include pharmaceutical substances and medications not obtained via personal prescription

Of the 324 participants who responded to questions about IDU in prison,^2^ 211 (65%) reported ever injecting in prison, 130 (40%) reported injecting during their current sentence and 81 (25%) reported injecting during a previous incarceration but not their current sentence. Among those reporting IDU during their current sentence, 60 (46%) reported injecting in the past month, reporting a median of eight injections over this period (IQR 2–28). The drugs most commonly injected during the current sentence were crystal methamphetamine (65%), buprenorphine-naloxone (45%) and heroin (35%).

### Alcohol and Other Drug Treatment Service Utilisation

Seventy-two percent (*n* = 288) of participants reported lifetime use of OST. Half (*n* = 143; 50%) of those ever on OST reported receiving OST at some point during their current sentence. Of the 121 who were receiving OST at the time of baseline interview, 95% were receiving methadone.

Most participants reported accessing some form of non-OST AOD treatment in the community (69%) or prison (75%) in their lifetime. The most commonly accessed community and prison-based non-OST AOD programs were detoxification services (45%) and group therapy (65%), respectively. Non-OST AOD treatment was accessed by 17% of participants in the 12 months before prison and 48% of participants during their current sentence. Over half of participants (54%) expressed a desire to access non-OST AOD services upon release from prison (Table 5).

**Table 5:** Non-opioid substitution therapy drug and alcohol treatment service utilisation at baseline

Non-OST treatment service type (multiple responses allowed	Ever accessed in community	Ever accessed in prison	Accessed during baseline incarceration	Wanted to access post-release
	*N* = 398	*N* = 399	*N* = 399	*N* = 393
	*n* (%)	*n* (%)	*n* (%)	*n* (%)
Any non-OST treatment	273 (69)	301 (75)	192 (48)	211 (54)
Group therapy	67 (17)	259 (65)	136 (34)	13 (3)
Individual counselling	121 (30)	123 (31)	52 (13)	166 (42)
Alcoholics or narcotics anonymous	79 (20)	85 (21)	37 (9)	28 (7)
Detox	179 (45)	NA	NA	10 (3)
Residential rehabilitation	121 (30)	NA	NA	20 (5)
One off information session	NA	190 (48)	82 (21)	NA
Other service type*	2 (1)	0 (0)	0 (0)	10 (3)

*Other service type includes naltrexone implant, drug court and voluntary urinary drug screens

## Discussion

Despite people with histories of incarceration and IDU typically exhibiting significant and complex health and social disadvantage [16, 19, 33] and exceptionally high rates of reincarceration [22], little is known about their experiences after release from prison. Our lack of understanding of individual, social and service access factors associated with patterns of drug use, health and criminogenic outcomes after release from prison impedes the development of effective policies and practices. The PATH Cohort Study purposively recruited people in prison for post-release follow-up who reported a history of regular IDU in the months immediately prior to incarceration and who were due for release within 12 weeks of interview. The PATH Cohort is unique in the Australian prisoner research context, where follow-up of prisoners has typically been much shorter [15, 34] or relied solely on secondary data linkage [9, 35]. The PATH Cohort is also unique in that it focuses on people who were injecting drugs regularly at the time of incarceration. This cohort represents a particularly complex and vulnerable group at high risk of a range of negative post-release outcomes. The detailed data collected at pre-release baseline, which we present in this paper, will couple with post-release follow-up data to provide unprecedented insights into the trajectories of this population over coming years to help inform pre- and post-release health and support programs.

The baseline characteristics of our cohort show expected indicators of socio-economic disadvantage, such as low educational attainment, accommodation instability and a reliance on government welfare and crime for income, and also an extensive history of incarceration that is reflected in community-recruited cohorts of PWID [31]. Our findings also show extensive patterns of intergenerational disadvantage. One quarter of respondents reported being removed from their parents as children, and almost half of participants who were parents or caregivers reported child protection involvement, and one in five reported having had their children removed from their care. While incarceration and intergenerational disadvantage has been extensively documented [36–38], the fact that almost two thirds of our cohort are the primary caregiver to a child underscores the substantial long-term social and economic costs associated with the nexus between disadvantage, dependent drug use and incarceration.

Substances reported as being injected by participants in the month prior to and during incarceration reflect recent trends in drug markets and drug-related harms in Australia and the emergence of methamphetamine as a commonly injected drug in Australia [2, 39]. In local Victorian drug trend surveillance, the proportion of PWID reporting methamphetamine as their most commonly injected drug increased from 17% in 2012 to 30% in 2016, although heroin remains the most commonly injected drug among survey respondents (66%) [40]. The over-representation of methamphetamine use in our cohort reflects other Australian law enforcement data on drug use among police arrestees [41, 42] and findings from a recent Australian study reporting methamphetamine as the most commonly injected drug prior to incarceration among prisoners with a history of IDU in New South Wales [43].

Among those who answered the question, almost two thirds of our sample reported ever injecting in prison (across a median of five previous incarcerations) and 40% reported injecting during their current sentence. While this prevalence of in-prison IDU is higher than other Australian studies, it is not unexpected given we purposively recruited participants frequently injecting prior to incarceration (at least monthly in the 6 months prior to incarceration) compared to others who recruited prisoners reporting any injecting in the 3 months pre-incarceration [42] or a lifetime history of injecting [23, 34]. The prevalence of pre-incarceration and in-prison methamphetamine injecting in our cohort, and trends in methamphetamine use among Australian PWID more broadly, highlights the need for effective methamphetamine treatment responses. Analysis of PWID cohort study data in Melbourne showed an association between drug first injected and current injecting drug preferences, and highlighted the need for flexible harm reduction and drug treatment services that respond to changing patterns of drug use [31]. This need for services that respond to changing drug use trends should also apply to law enforcement responses and programs available in prison. While OST is widely available in Victorian prisons (and was the most commonly accessed prison drug treatment program in our cohort), prison programs for methamphetamine dependence have relied primarily on group and individual counselling. While counselling treatment approaches to methamphetamine dependence have demonstrated some success in community settings [44], their effectiveness in prison settings is yet to be established [45]. A forthcoming trial of pharmacotherapy for the management of methamphetamine dependence [46] may result in new drug treatments suited to implementation in prison.

Our experience recruiting cohort participants provides insights into non-random and targeted prison recruitment strategies. Targeting recruitment to recent and frequent IDU meant that screening for eligibility via prison administrative data was not possible. Instead, we relied mostly on researchers spending extensive time in prisons and building trust and rapport with prisoners. Our recruitment strategies initially relied upon promoting the study via posters and program workers, but were soon modified to focus more on researchers’ direct engagement with prisoners in their units or at clinical or program visits. The ability to communicate the purpose of the study and answer questions about participation on the spot, and to encourage information about the study spreading via word-of-mouth, meant that direct engagement strategies were the most fruitful by far. In the context of aiming to recruit participants close to their expected release date, the most significant recruitment challenge was the transfer of eligible and interested prisoners out of recruitment prisons before scheduled interviews could take place. Movement between prison sites late in a sentence is also a challenge for the provision of effective transitional support for people leaving prison, particularly if transitional support service providers differ according to prison region or site.

Our study has limitations associated with sampling and reporting. First, as noted above, our eligibility criteria and limitations in administrative data meant that we were unable to implement a random sampling or consecutive sampling recruitment approach, and as such, findings may not be generalizable to the broader Victorian prison population with histories of IDU. More than twice the number of people recruited submitted an expression of interest form to participate in the study. While a small number were excluded from the study because they did not meet eligibility criteria (e.g. pre-incarceration drug use, release dates beyond the study recruitment period), inability to participate in the study mostly occurred because of transfers to other prisons prior to individuals becoming eligible to complete baseline surveys on the basis of expected release date. Movements between prisons overwhelmingly occur due to operational requirements rather than being based on prisoner behaviours or characteristics, and this inability to participate is therefore not expected to result in meaningful sampling bias. The restriction of the study to three recruitment sites and the variation in numbers recruited at each site may also introduce some recruitment bias. This paper is mostly based on self-reported responses and may be susceptible to reporting and recall bias, particularly for questions that refer to participants’ pre-incarceration experiences. However, for most survey domains, future record linkage will provide objective data to validate self-report and other measures (e.g. lifetime IDU history) that could not be collected in any other way. While our sample includes a higher proportion of Indigenous participants (17%) relative to the general Victorian prison population (7.8%) [12], it is unclear the extent to which this represents an over-representation of prisoners with IDU histories. The sample also includes participants with a median sentence length of 206 days. In Victoria, 25.8% of prisoners have an effective sentence length of less than 1 year. The shorter sentence length in our sample may reflect the types of crimes committed by people incarcerated for drug-related offences. Shorter sentences in this group bear further exploration in future analysis given access to some prison health programs (e.g. hepatitis C treatment) is restricted to those on longer sentences or residing in non-remand prisons. Finally, incarcerated women with IDU histories were not recruited and the study will therefore not reflect the experiences and specific challenges faced by women after release from prison [47]. The original study design included an over-sample of 100 female prisoners; however, operational pressures at the women’s prisons in Victoria precluded their recruitment (e.g. dealing with an influx of women prisoners and engaging in substantial new construction).

Our description of the baseline characteristics of PATH participants shows a cohort with substantial challenges with respect to physical and mental health, drug use, individual family histories, history of offending, educational attainment and employment history. Instability of accommodation and state involvement in the care of children further demonstrate the complexity of providing adequately coordinated, holistic care and support for this population during their reintegration into the community. Future analyses of prospective data will identify unmet community needs and describe the time-variant and time-invariant factors associated with specific health, drug use and criminogenic trajectories in this population. We aim to provide novel information to support policy and practice change related to the timing, targeting and modes of interventions designed to improve this population’s health and social outcomes.

## Footnotes

1 While recruitment occurred solely within adult prisons, individuals who are under 18 years are occasionally detained within these settings.

2 These were considered by participants to be sensitive questions and a number of participants declined to answer them.
